# MR-based radiomics predictive modelling of EGFR mutation and HER2 overexpression in metastatic brain adenocarcinoma: a two-centre study

**DOI:** 10.1186/s40644-024-00709-4

**Published:** 2024-05-21

**Authors:** Yanran Li, Yong Jin, Yunling Wang, Wenya Liu, Wenxiao Jia, Jian Wang

**Affiliations:** 1 Department of Radiology, The First Affiliated Hospital of Xinjiang Medical University, Urumqi, Xinjiang 830054 China; 2 Department of Radiology, Changzhi People’s Hospital, Changzhi, 046000 Shanxi Province China

**Keywords:** T1-CE brain MR sequences, Adenocarcinoma, Brain metastasis, Radiomics, Gene expression

## Abstract

**Objectives:**

Magnetic resonance (MR)-based radiomics features of brain metastases are utilised to predict epidermal growth factor receptor (EGFR) mutation and human epidermal growth factor receptor 2 (HER2) overexpression in adenocarcinoma, with the aim to identify the most predictive MR sequence.

**Methods:**

A retrospective inclusion of 268 individuals with brain metastases from adenocarcinoma across two institutions was conducted. Utilising T1-weighted imaging (T1 contrast-enhanced [T1-CE]) and T2 fluid-attenuated inversion recovery (T2-FLAIR) sequences, 1,409 radiomics features were extracted. These sequences were randomly divided into training and test sets at a 7:3 ratio. The selection of relevant features was done using the least absolute shrinkage selection operator, and the training cohort’s support vector classifier model was employed to generate the predictive model. The performance of the radiomics features was evaluated using a separate test set.

**Results:**

For contrast-enhanced T1-CE cohorts, the radiomics features based on 19 selected characteristics exhibited excellent discrimination. No significant differences in age, sex, and time to metastasis were observed between the groups with EGFR mutations or HER2 + and those with wild-type EGFR or HER2 (*p* > 0.05). Radiomics feature analysis for T1-CE revealed an area under the curve (AUC) of 0.98, classification accuracy of 0.93, sensitivity of 0.92, and specificity of 0.93 in the training cohort. In the test set, the AUC was 0.82. The 19 radiomics features for the T2-FLAIR sequence showed AUCs of 0.86 in the training set and 0.70 in the test set.

**Conclusions:**

This study developed a T1-CE signature that could serve as a non-invasive adjunctive tool to determine the presence of EGFR mutations and HER2 + status in adenocarcinoma, aiding in the direction of treatment plans.

**Clinical relevance statement:**

We propose radiomics features based on T1-CE brain MR sequences that are both evidence-based and non-invasive. These can be employed to guide clinical treatment planning in patients with brain metastases from adenocarcinoma.

**Supplementary Information:**

The online version contains supplementary material available at 10.1186/s40644-024-00709-4.

## Introduction

Cell surface receptor tyrosine kinases, such as the epidermal growth factor receptor (EGFR) and human epidermal growth factor receptor 2 (HER2), dimerise with other HER family receptors to transmit growth signals [1]. Many common cancers often involve the activation, overexpression, or mutation of EGFR and HER2. When EGFR and HER2 are overexpressed in tumour cells, these cells multiply rapidly [2]. Numerous solid tumours have been found to harbour mutant forms of EGFR, with these mutations being associated with tumour progression, drug resistance, and patient survival [3]. In certain individuals with various solid tumours, HER2 is overexpressed [4], substantially predicting poorer overall survival. Recent studies have identified HER2 kinase domain mutations in 4% of all primary lung cancer tumours and 10% of those with adenocarcinoma histology [5]. As a result, timely determination of EGFR mutations and HER2 overexpression is crucial for predicting therapeutic response and determining specific treatment plans.

The currently accepted method for determining genetic status is biopsy. However, its highly invasive nature often precludes the collection of tissue samples from the main tumour or brain metastases. The size of the lesion, degree of intratumoral cystic necrosis, degree of paratumoral oedema, and degree of imaging signal are all characteristics of brain metastasis that may be effectively reflected by magnetic resonance (MR) imaging [6, 7]. Nonetheless, MR imaging is unable to predict alterations at the molecular level. Therefore, precise, non-invasive quantitative approaches are required to provide a real-time complement to histologic evaluation and represent intratumoral heterogeneity.

Existing brain metastasis radiomic research has primarily focused on building models to differentiate between various primary malignancies or lung cancer subtypes [8–11]. However, there has been limited information available on the radiomic characteristics of brain metastases. Brain metastases might offer a genetic opportunity to select patients for specific treatments.

This study explores the potential of radiomics in analysing EGFR and HER2 gene statuses in patients with brain metastases. It aims to create a relevant model using radiomics for this purpose and verify the model’s validity. Such a model could be clinically guided by radiomics for treatment planning in patients with brain metastases from adenocarcinoma.

## Methods and materials

### Study content

**Fig. 1:**
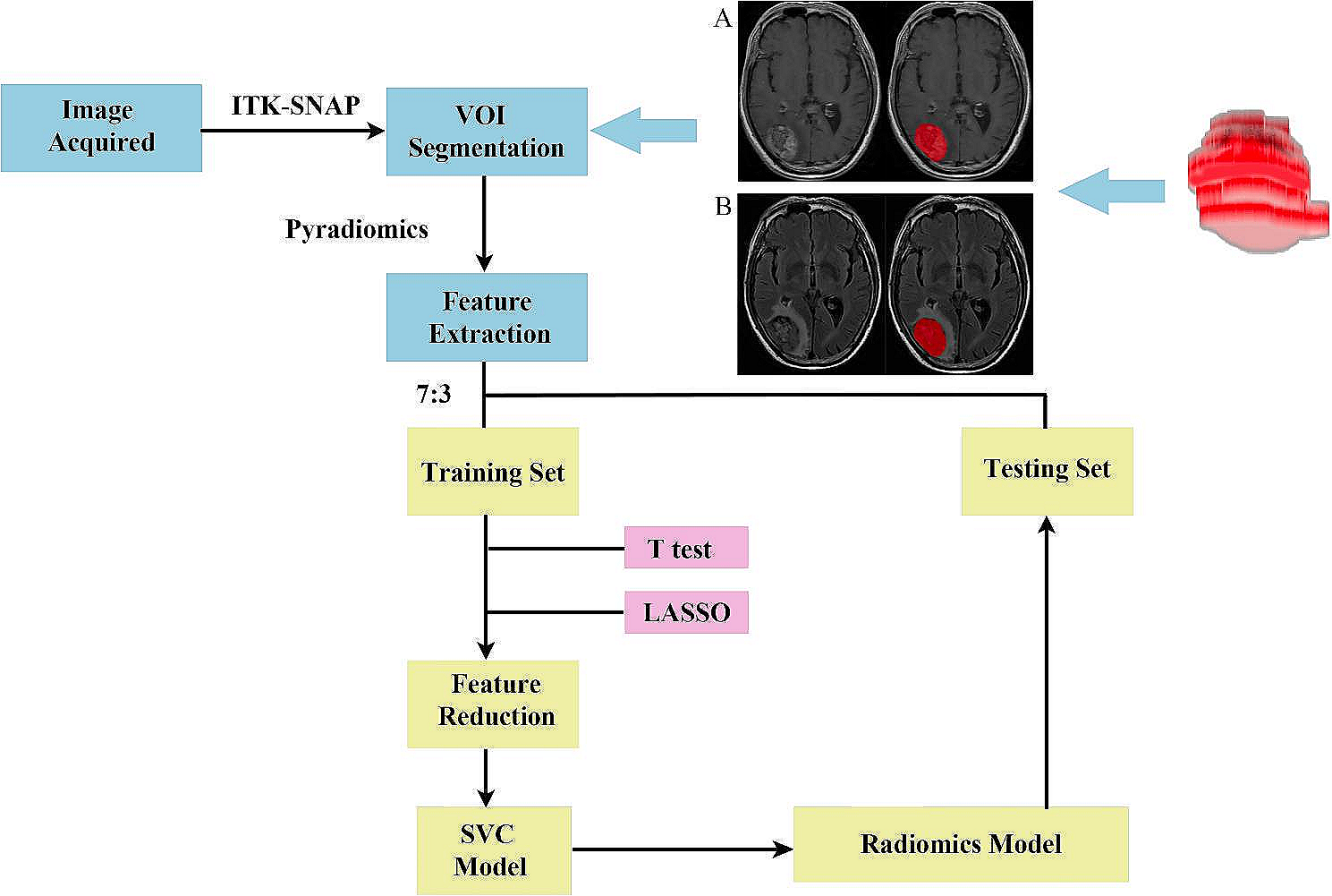
Workflow for radiomics modeling and analysis including segmentation of metastases and examples from MR images

This study aims to utilise T1 contrast-enhanced (T1-CE) and T2 fluid-attenuated inversion recovery (T2-FLAIR) radiomic features to predict EGFR mutations and HER2 overexpression in adenocarcinoma in a personalised manner. Radiological features were derived from segmented regions of the MRI images. The collected data were divided into training and test sets according to the experimental design and were subsequently used for training and independent validation of the model, respectively. The most valuable radiological features were selected using the least absolute shrinkage and selection operator (LASSO) technique. The prediction model was developed using the support vector classifier (SVC) method. A detailed description and analysis workflow can be seen in Fig. 1.

### Participants

Between January 2015 and January 2022, this two-centre retrospective cohort study was authorised by our hospital’s ethics committee. Patients were initially diagnosed with brain metastases resulting from cancer using MR imaging and genetic testing. Pathology confirmed the diagnosis of all primary lesions. All MR-confirmed metastases were reported independently by nuclear medicine specialists and radiologists with at least five years of board certification.

To be included in the study, patients had to meet the following criteria: (1) primary tumour pathologically confirmed as adenocarcinoma by surgery or biopsy, (2) presence of brain metastases, (3) brain metastases confirmed by pathology, and (4) assessment using MR imaging. The exclusion criteria were as follows: (1) various types of original malignancies, (2) absence of genetic testing, (3) patients who had received systemic therapy, and (4) lack of follow-up data.

### EGFR mutation status and overexpressed HER2

All adenocarcinoma patients underwent an evaluation of their EGFR status using next-generation sequencing technologies.

The HER2 status of all patients with adenocarcinoma was assessed using immunohistochemistry (IHC) or fluorescence in situ hybridisation (FISH) [12]. Two independent pathologists reviewed the patients’ IHC results at the time of the HER2 status assessment. Patients were considered negative for HER2 if IHC results were 0/1 + and positive if IHC results were 3+. HER2 status was further determined using FISH results when IHC results were 2+ [12, 13].

### MR image acquisition

Brain MR images were obtained from patients with adenocarcinoma at their initial diagnosis of brain metastases. At the First Affiliated Hospital of Xinjiang Medical University, patients underwent evaluation using a 3.0 T MR scanner (MAGNETOM Skyra, Siemens Healthineers, USA) and a 3.0 T MR scanner (Discovery MR750, GE Healthcare, USA). At Changzhi People’s Hospital, a 1.5 T MR scanner and a 3.0 T MR scanner (MAGNETOM Verio, Siemens Healthineers, USA) were employed. The T1-CE and T2-FLAIR sequences, which are commonly used for detecting brain metastases, were chosen for the extraction of image features [7, 14–17]. The image matrix size varied from 240 × 240 to 256 × 256 pixels, with slice thickness ranging from 1 mm to 6 mm. Additional scanning parameters for these sequences are presented in Supplementary Table 1.

### Image segmentation

Regions of interest (ROIs) on the MR images of the aforementioned sequences were delineated using ITK-SNAP [18] around the metastases. To minimise the impact of clustering, only one ROI per image was selected. Initially, ROIs were created around each axial section of the metastatic profile to examine tumour heterogeneity. Subsequently, areas of oedema, haemorrhage, cystic changes, and necrosis were excluded from the images. Representative sections of the metastases for each sequence were chosen. The red spots in Fig. 1 indicate the axial distribution of the metastases.

### Radiomics features extraction

Before feature extraction, several preprocessing steps were undertaken to enhance texture recognition. First, the bin width was set to 25. Second, Z-score normalisation was applied to the MR images. Finally, cubic interpolation was utilised to resample the ROIs isotropically to an in-plane resolution of 3 × 3 × 3 mm, ensuring the consistency of proportions and orientation in the acquired 3D features [19].

Prior to radiomic feature extraction, each image underwent preprocessing with eight image filters to highlight specific details and reduce noise. These filters, comprising Wavelet (HH, HL, LL, LHH, LLL), Laplacian of Gaussian (sigma = 1, 2, 3), Square, Square Root, Logarithm, Local Binary Pattern, Gradient, and Exponential, were used to evaluate three different categories of radiomic features in each image. A total of 1,409 radiomic features were extracted per tumour, with an equivalent number of features generated from both T1-CE and T2-FLAIR sequences. The radiomic features were extracted using the open-source Python program Pyradiomics (https://github.com/Radiomics/pyradiomics). Various radiomic features were utilised for this extraction process.

### Establishment of radiomics features

Data from Institutions 1 and 2 were combined and subsequently divided into training (70%) and test (30%) sets after manually segmenting and extracting features from all ROIs for each participant. A PyRadiomics-based pipeline, previously tested, was used to extract radiomics features and conduct feature selection. Among the radiomics features retrieved, there were several duplicated, unstable, and irrelevant imaging characteristics. Feature selection methods were used to identify and select the most informative, stable radiomics features. This approach also helps prevent overfitting. The final feature set was established using the LASSO method and recursive feature elimination.

### Model building

A radiomics model was constructed using a linear SVC based on these two MR image sequences after feature selection. To address the data imbalance, the synthetic minority oversampling technique algorithm with default parameters was applied [20]. This created minority instances along a line connecting a minority instance to its nearest neighbours.

### Interobserver reproducibility evaluation

In our radiomics inquiry, ROI segmentation repeatability and radiomics feature extraction reproducibility are integral to the interobserver reproducibility process. The repeatability of the ROI segmentation was tested through multi-reader segmentation. For this purpose, two independent radiologists manually delineated ROIs in four MR sequences using the same annotation program, ITK-SNAP (http://www.itksnap.org). For reproducible radiomics feature extraction, we employed PyRadiomics, an open-source program, to compute the radiomics features and to perform image preprocessing. The feature extraction process was guided by a.yaml file, allowing separate individuals to replicate the extraction of radiomics features outlined in our study using the same file.

Pleas einclude the supplier’s information.

### Statistical analysis

SPSS (version 22.0) was utilised for the statistical analysis. To explore group differences, we applied chi-squared tests to categorical variables and t-tests for continuous data. The model’s effectiveness was assessed using the receiver operating characteristic (ROC) curve and the area under the curve (AUC). Sensitivity and specificity metrics were calculated using a threshold of 0.5. A p-value of less than 0.05 was deemed statistically significant.

## Results

### Patients’ information

**Fig. 2:**
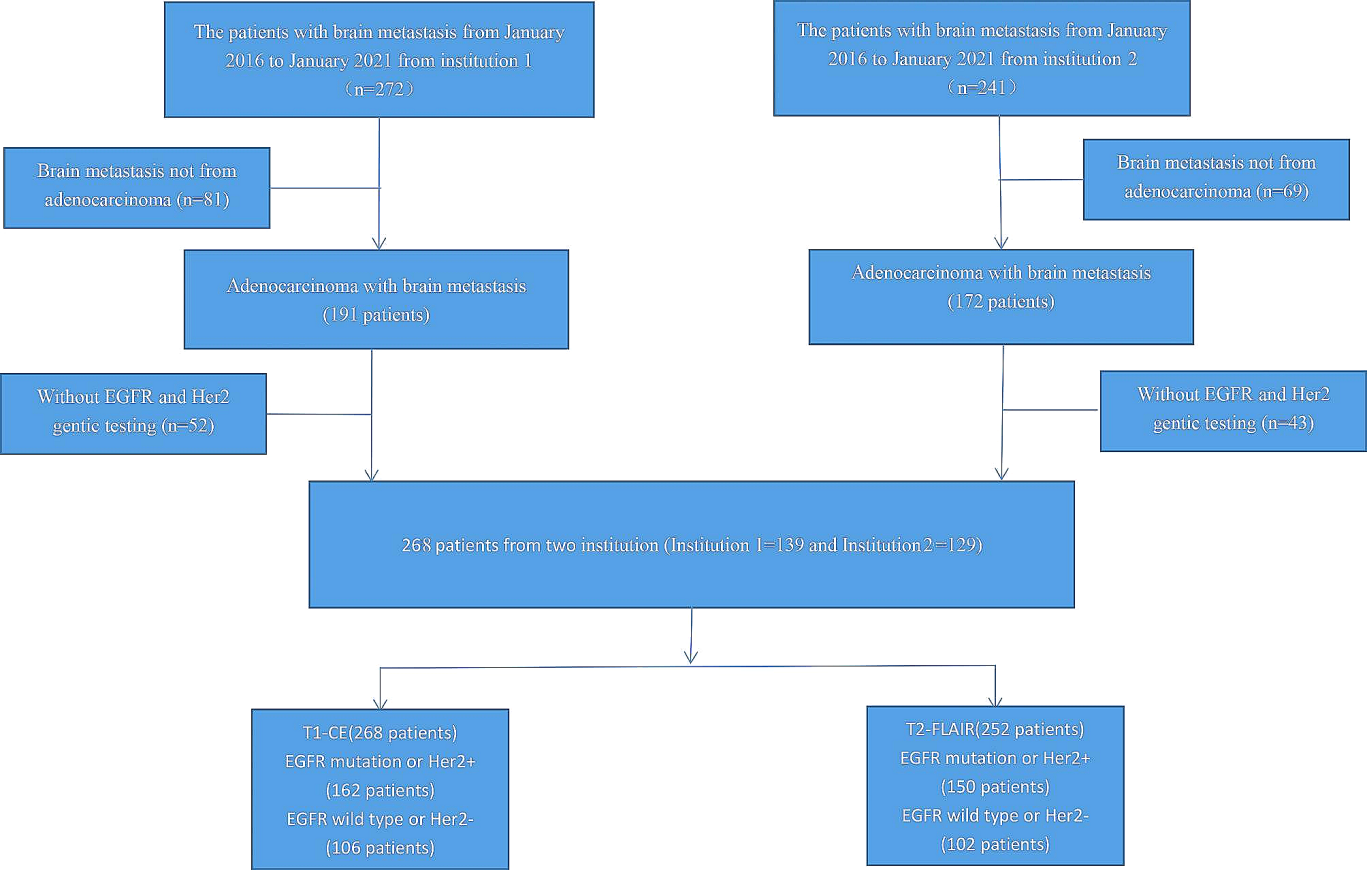
The flow chart of patient enrollment

Between January 2015 and January 2022, we conducted a retrospective analysis of 513 patients with brain metastasis from two institutions (Institution 1 = 272, Institution 2 = 241). In Institution 1, 81 patients and in Institution 2, 69 patients with brain metastasis were excluded as these metastases did not originate from adenocarcinoma. Additionally, 52 patients in Institution 1 and 43 in Institution 2 were excluded due to a lack of genetic testing on these brain metastases. Ultimately, 268 patients with documented T1-CE sequences in hospital archives were enrolled across both institutions (Fig. 2). The gene expression features of these 268 patients are summarised in Table 1. Overall, there were no significant differences in age, sex, and time to metastasis between the groups with EGFR mutant or HER2 + and those with wild-type EGFR or HER2− (*p* > 0.05).

**Table 1:** Pathological diagnosis of primary tumors

Pathological diagnosis of primary focus	Gene expression				
	EGFR mutation	HER2+	EGFR wild-type	HER2-	Total
Lung adenocarcinoma	105 (39.2%)		56 (20.9%)		161 (60.1%)
Breast adenocarcinoma	8 (3%)	24 (9%)		22 (8.2%)	54 (20.1%)
Gastric adenocarcinoma		9 (3.4%)		7 (2.6%)	16 (6%)
Adenocarcinoma of colon			6 (2.2%)	8 (3%)	14 (5.2%)
Undefined adenocarcinoma	16 (6%)		7 (2.6%)		23(8.6%)
Total	129 (48.1%)	33 (12.3%)	69 (25.8%)	37 (13.8%)	268

### Selection of features and creation of radiomics features

**Fig. 3:**
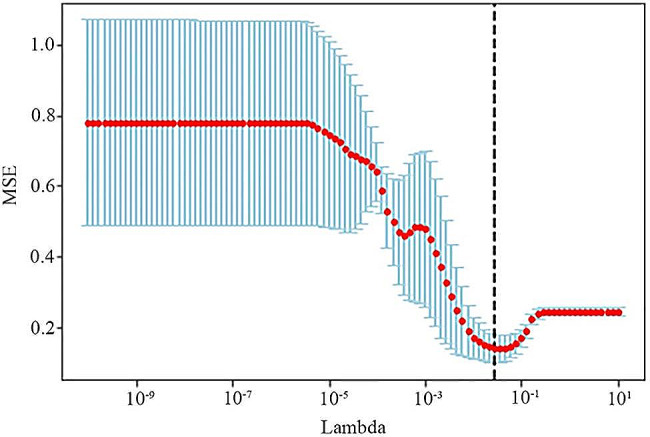
The MSE versus Lambda plot for T1-CE

**Fig. 4:**
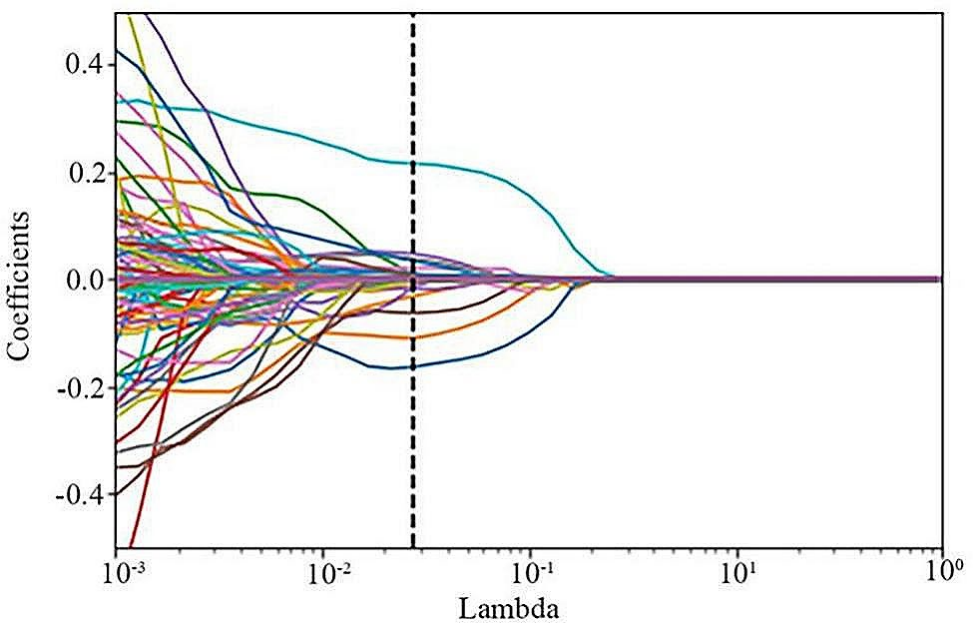
The LASSO coefficients versus Lambda for T1-CE

For a more robust feature selection, we employed a combination of cross-validation and grid search techniques. The search range consisted of 100 evenly spaced values within the range [− 10, 1] (lambda). After examining these values, 0.027826 emerged as the optimal lambda value for T1-CE. Both the mean squared error (MSE) versus lambda plot and the LASSO coefficients versus lambda plot were provided. Figure 3 illustrates the MSE vs. lambda plot, whereas Fig. 4 displays the LASSO coefficients versus the lambda plot.

A total of 19 out of 1,409 considered radiomics features were ultimately selected for the T1-contrast sequence. The chosen radiomics features for the T1-contrast sequence included original_firstorder_InterquartileRange, original_firstorder_Range, original_glszm_SmallAreaEmphasis, original_glszm_SmallAreaLowGrayLevelEmphasis, wavelet-LLH_firstorder_Maximum, wavelet-LLH_glcm_ClusterProminence, wavelet-LLH_glcm_ClusterShade, wavelet-LLH_glszm_SmallAreaEmphasis, wavelet-LLH_ngtdm_Contrast, wavelet-LHL_firstorder_Mean, wavelet-LHH_firstorder_RobustMeanAbsoluteDeviation, wavelet-LHH_glrlm_LongRunHighGrayLevelEmphasis, wavelet-LHH_glszm_SizeZoneNonUniformityNormalized, wavelet-HLH_glszm_SizeZoneNonUniformityNormalized, wavelet-HLH_ngtdm_Complexity, wavelet-HHL_gldm_HighGrayLevelEmphasis, lbp-2D_firstorder_MeanAbsoluteDeviation, logarithm_glszm_SmallAreaEmphasis, and logarithm_glszm_SmallAreaLowGrayLevelEmphasis.

### Radiomics features performance

After feature selection, a radiomics feature set was created using an SVC model to differentiate between mutant EGFR and HER2 + and wild-type EGFR and HER2. For the MR sequences, a radiomics feature set was developed using the training cohort, and the test set was employed to evaluate the feature set’s performance. Its complete performance is detailed in Table 2.

**Table 2:** Performance of each MR sequence’s radiomics signature in the training group and the testing cohort

Metrics	Training cohort		Testing cohort	
	T1-CE	T2-FLAIR	T1-CE	T2-FLAIR
AUC	0.98	0.86	0.82	0.70
ACC	0.93	0.74	0.70	0.62
SPE	0.93	0.67	0.69	0.67
SEN	0.92	0.83	0.73	0.57

AUC area under the curve, ACC classification accuracy, SEN sensitivity, SPE specificity

The radiomics signature of T1-CE demonstrated the best performance in the training group. Analysis of radiomics profiles for T1-CE revealed an AUC of 0.98, classification accuracy (ACC) of 0.93, sensitivity (SEN) of 0.92, and specificity (SPE) of 0.93. This performance was also evaluated on a separate test set, where the T1-CE radiomics signature showed an AUC of 0.82, ACC of 0.70, SEN of 0.73, and SPE of 0.69, indicating strong performance.

**Fig. 5:**
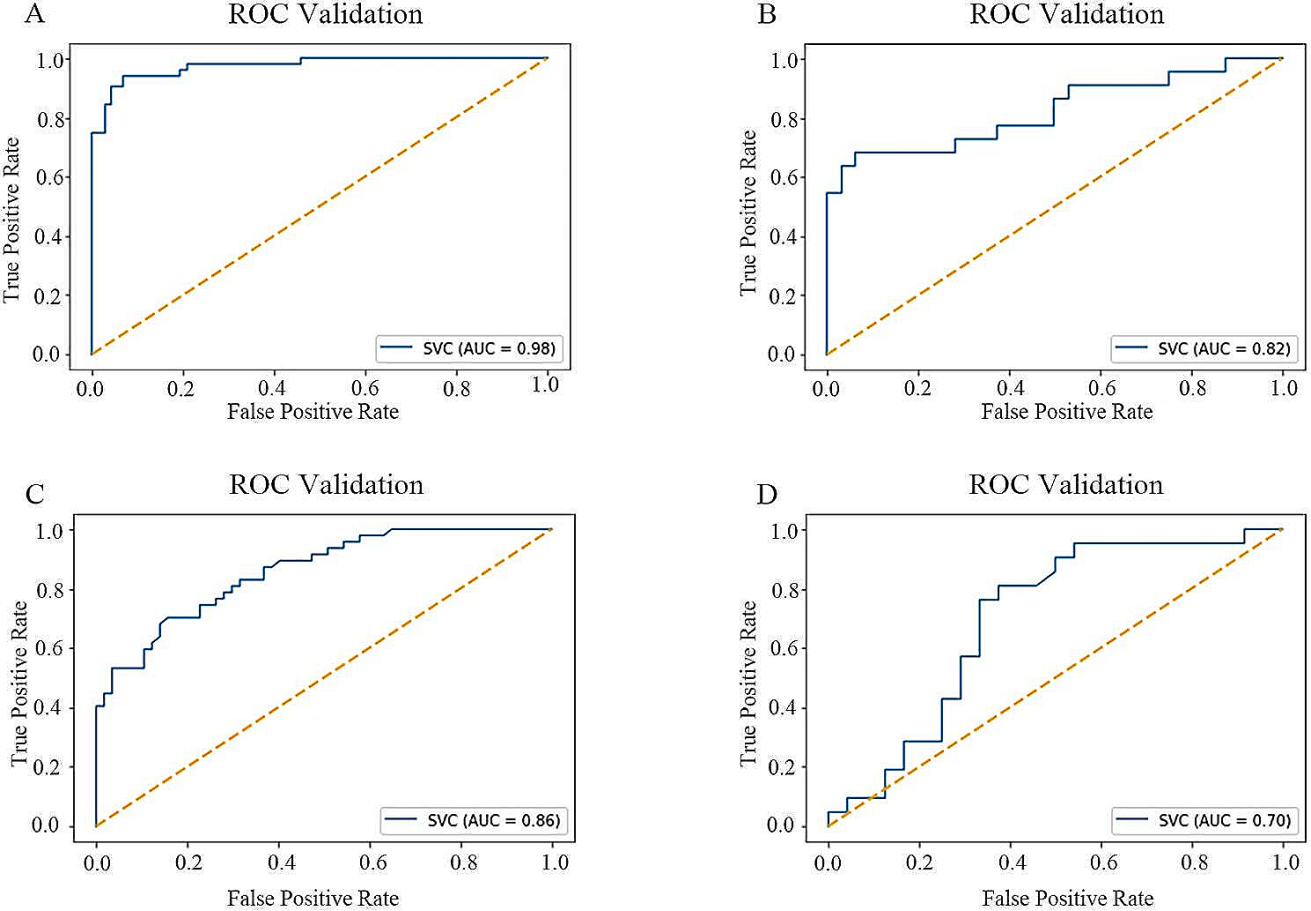
ROC curves in the training and testing cohort for various MR sequences. A.T1-CE training cohort. B. T1-CE testing cohort. C. T2-FLAIR training cohort. D. T2-FLAIR testing cohort

In contrast, the T2-FLAIR sequence exhibited substantially weaker performance. The radiomics signature of T2-FLAIR in the training group achieved an AUC of 0.86, ACC of 0.74, SEN of 0.83, and SPE of 0.67. In the test set, the AUC of the T2-FLAIR was 0.70. Figure 5 illustrates the ROC curves for these two MR sequences. Additionally, the AUC scores associated with different radiomics interpolators are presented in Supplementary Fig. 1.

## Discussion

At present, invasive biopsy or surgical resection of brain metastases is typically required to identify gene mutation status in clinical practice. However, these invasive examinations may lead to serious complications, such as postoperative bleeding and cerebrospinal fluid leakage. Furthermore, it is challenging to obtain accurate gene mutation statuses for patients who cannot tolerate surgery [21]. The imageomics model created in this study demonstrates high accuracy in predicting gene mutations in adenocarcinoma brain metastases and possesses substantial predictive power. Consequently, this study provides a potent non-invasive tool for predicting EGFR gene mutation status and HER2 overexpression in adenocarcinoma brain metastases, thereby greatly enhancing patient prognosis. Additionally, prior studies have primarily focused on the prediction of gene mutations in lung cancer brain metastases, with the research scope being somewhat limited. Most of these studies target EGFR mutations, with fewer investigations into other gene mutations [22]. Imageomics studies predicting EGFR mutation status in patients with lung cancer typically rely on CT or MRI images of primary lung lesions [23]. In contrast, most MRI imageomics studies of brain metastases aim to distinguish between different lung cancer subtypes or primary sites [24], with a limited number focused on predicting EGFR mutation status in lung cancer. Sui et al. [25] predicted the EGFR mutation status in primary lung adenocarcinoma based on MRI imaging omics characteristics of brain metastases, exploring the optimal MRI sequence for EGFR mutation prediction. Their results indicated that imaging omics characteristics based on multiple combined MRI sequences (enhanced T1WI, FLAIR, and DWI) could serve as a non-invasive auxiliary tool for predicting EGFR mutation status in lung adenocarcinoma. Jiang et al. [26] established a 3D-T1WI enhanced imaging omics model of brain metastases from non-small cell lung cancer to assess its diagnostic efficacy for EGFR mutation status in these patients. The findings showed that 3D-T1WI enhanced imaging omics of brain metastases from non-small cell lung cancer had high diagnostic efficacy and net clinical benefit in determining EGFR mutation status. This study’s establishment of an imageomics model to predict gene mutation in brain metastases and the identification of effective MRI diagnostic sequences enrich the research in this field and provide a novel diagnostic method that can accurately predict EGFR mutation/HER2 + status in adenocarcinoma.

Ahn utilised radiomics features to identify EGFR mutations in the treatment of primary lung cancer and brain metastases, achieving a prediction AUC of 86.81 [27]. We examined 268 patients using the T1-CE sequence and 252 using the T2-FLAIR sequence. Higher rates of EGFR mutation were observed in women than in men, with the majority of changes being prevalent [28, 29]. Clinical factors such as age, sex, smoking, and drinking cannot effectively predict tumour mutation status. Meanwhile, multiple studies have demonstrated that classic imaging characteristics, including breast density [30], a spiculated mass [31], and microcalcifications [32] on mammograms, are strongly correlated with HER2 status. However, the predictive capability of these characteristics for HER2 status is limited.

In establishing HER2 status, challenges include the scarcity of sufficient tissue samples for genetic testing, the high cost of genetic detection, and the extended duration required to obtain genetic testing results. To address these challenges, our study measured heterogeneity within MR images by exploring the potential correlation between the spatial distribution of voxel grey levels and the shape of the underlying tissue. These methods may provide additional insights into the adenocarcinoma phenotype and genotype, thereby improving the prediction of related gene statuses.

Nineteen key characteristics, all statistically significant, were selected from 1,409 candidate features extracted from normalised MR imaging data. We then developed a radiomics signature using SVC regression on the selected variables, and its efficacy was evaluated in the test set. This proposed radiomics signature demonstrates high accuracy in differentiating between mutant EGFR or HER2 + and normal EGFR or HER2−.

Previous research indicates that radiomics analysis is effective in determining the status of EGFR mutations [33–35]. Furthermore, the radiomics signature of T2-FLAIR provided an AUC of 0.7, ACC of 0.62, SEN of 0.57, and SPE of 0.67. These findings suggest that using T1-CE data, the proposed radiomics signature can predict EGFR mutation/HER2 + status non-invasively. However, the T2-FLAIR sequence exhibits limited discriminatory ability for classifying EGFR mutation status.

Associating radiomics properties with clinical imaging is an emerging field that enhances molecular biology relevance. The correlation between radiomics traits and molecular characteristics may reflect gene expression variations within and across tumours. Tumours enriched in genetic mutations during development lead to alterations in downstream signalling pathways, causing widespread biological responses and driving processes such as cell proliferation and inflammation. Extracting a broad array of quantitative imaging metrics, proven effective in other studies, can translate these changes into interpretable data. Segal et al. were the first to demonstrate the use of radiogenomic maps, reconstructing tumour gene expression patterns using 28 imaging characteristics [36]. Koay et al. posited that quantifiable imaging parameters could reveal genetic and pathological tumour heterogeneity. They observed that patients with prominent tumours showed substantially less stroma, more mesenchymal features, and a higher likelihood of multiple signalling pathway alterations [37]. Grossmann et al. [38] suggested that radiomics facilitates the non-invasive assessment of adenocarcinoma molecular and clinical traits, hypothesising a link between radiomic imaging properties and signalling pathways. These studies demonstrate that image phenotypes can be used for non-invasive assessments with therapeutic implications and may indicate the activity of different biological pathways.

In our study, we established a connection between the resilience of the radiomics signature, its enhanced performance, and the tumour’s molecular biological characteristics. Lung cancer development and progression are influenced by the transmembrane protein EGFR, which has cytoplasmic kinase activity. EGFR promotes cell proliferation upon binding to specific ligands [39]. Aberrant EGFR expression, such as gene amplification, overexpression of EGFR ligands, and mutations, all contribute to tumour growth and metastasis [3]. Experimental models using animal models with tumour cell xenografts have shown that blocking EGFR increases tumour cell mortality, reduces angiogenic factor production, and prolongs survival [3].

It has been demonstrated that HER2, through heterodimerisation with EGFR and other HER family members, activates a wide array of intracellular signalling pathways. Notably, HER2 appears to be the preferred heterodimerisation partner for other members of the HER family [40]. When HER2 is overexpressed, a substantial number of HER2 homodimers and heterodimers are formed. This aberrant downstream signalling is partly mediated by the constitutive activation of the PI3-kinase/Akt and ras/mitogen-activated protein kinase pathways. These pathways inhibit apoptosis, promote cell motility, and support cell growth and survival. Transfecting HER2 into human tumour cell lines has been shown to create a more aggressive tumour cell phenotype [41].

However, there are several limitations to this research. Since the amount of segmentation determines the radiomics characteristic, segmentation is initially one of the more challenging aspects of radiomics. To ensure the consistency of ROI delineation, we aim to establish a deep-learning segmentation model based on large datasets in future work. Second, the information was obtained retrospectively, introducing a potential selective bias that cannot be eliminated. The participants in this study also originated from two separate institutions, each with its own set of protocols and scanners, resulting in notable variations in patients’ characteristics between the two centres. A normalisation strategy was employed to mitigate data discrepancies across multiple centres and enhance the stability of features and various models.

## Conclusion

In conclusion, we have proposed a radiomics signature derived from T1-CE brain MR sequences that are both reliable and non-invasive. Our brain metastasis model has the potential to more accurately predict EGFR mutation/HER2 + status in patients with adenocarcinoma compared with models predominantly focused on primary lesions. This could substantially assist in directing individualised treatment regimens. While larger prospective studies are necessary to validate our findings, our initial results indicate that radiomics could serve as a non-invasive tool for tumour genotyping through the examination of metastases.

### Electronic supplementary material

Below is the link to the electronic supplementary material.

Supplementary Material 1

Supplementary Material 2

## Data Availability

All data generated or analyzed during this study are included in this published article.

## Abbreviations

ACC: Accuracy
AUC: Area Under Curves
CRC: Colorectal Cancer
EGFR: Epidermal Growth Factor Receptor
GLCM: Gray Level Co-occurrence Matrix
GLRLM: Gray Level Dependence Matrix
GLSZM: Gray Level Size Zone Matrix
GLDM: Gray Level Dependence Matrix
HER2: Human Epidermal Growth Factor Receptor 2
LASSO: Least Absolute Shrinkage and Selection Operator
linear SVC: Linear Support Vector Classifier
MAP: Mitogen-Activated Protein
NGS: Next-Generation Sequencing
NGTDM: Neighboring Gray Tone Difference Matrix
NSCLC: Non-Small-Cell Lung Cancer
OS: Overall Survival
ROI: Region of Interest
SCCHN: Squamous Cell Carcinoma of the Head and Neck
SEN: Sensitivity
SMOTE: Synthetic Minority Oversampling Technique
SPE: Specificity
T1-CE: Contrast-Enhanced T1-Weighted Imaging
T2-FLAIR: T2 Fluid-Attenuated Inversion Recovery
TKs: Tyrosine Kinases
