# Supplementary figures and images for: MR-based radiomics predictive modelling of EGFR mutation and HER2 overexpression in metastatic brain adenocarcinoma: a two-centre study
Source: Cancer Imaging. 2024 May 21;24:65. doi: 10.1186/s40644-024-00709-4 (PMC11110398; doi:10.1186/s40644-024-00709-4)

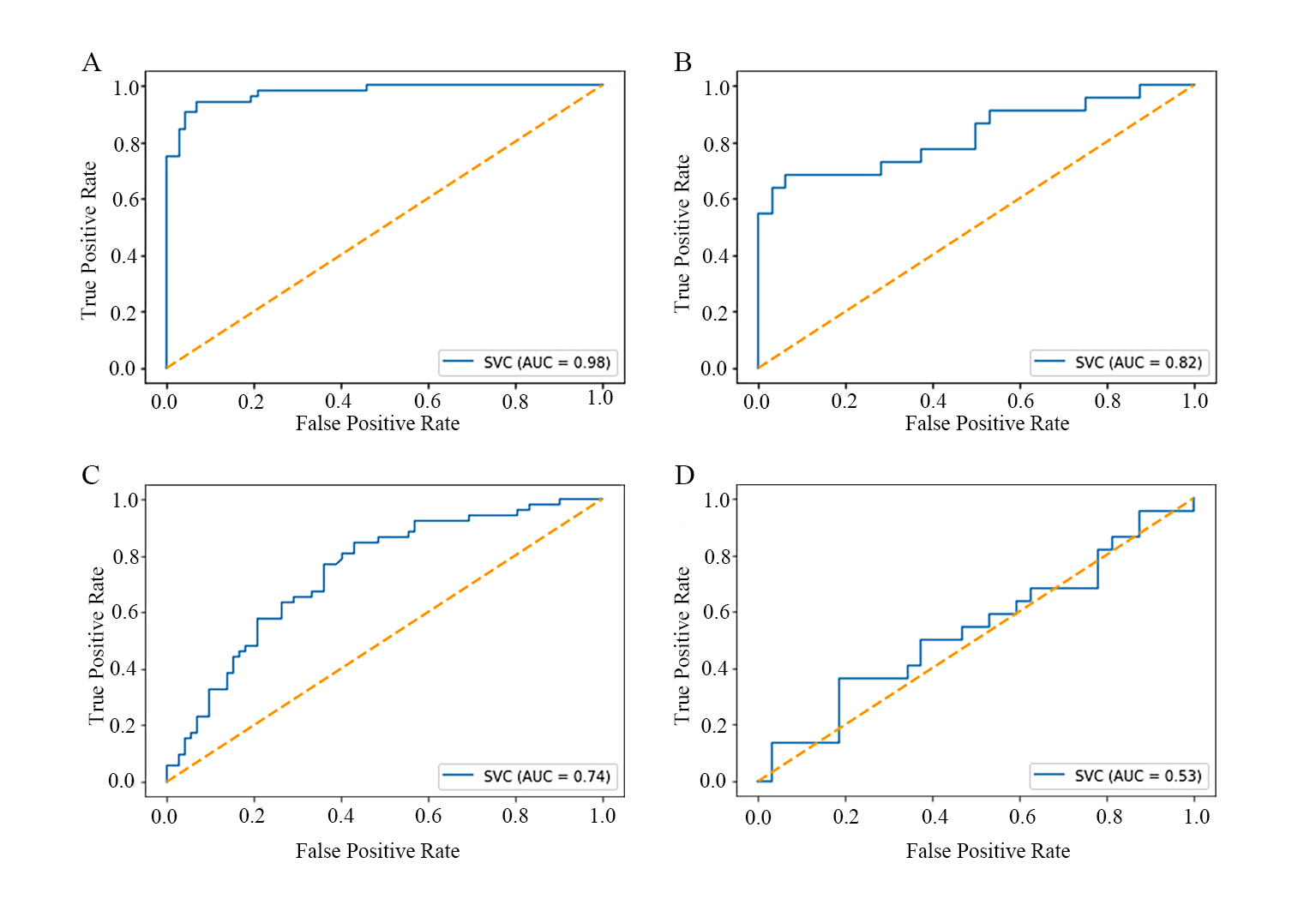

Supplement: Supplementary file 1 — Supplementary Material 1 [file 40644_2024_709_MOESM1_ESM.jpg]
