# Supplementary material for: MR-based radiomics predictive modelling of EGFR mutation and HER2 overexpression in metastatic brain adenocarcinoma: a two-centre study
Source: Cancer Imaging. 2024 May 21;24:65. doi: 10.1186/s40644-024-00709-4 (PMC11110398; doi:10.1186/s40644-024-00709-4)
Supplement: Supplementary file 2 — Supplementary Material 2 [file 40644_2024_709_MOESM2_ESM.docx]

| Scanner Type | Sequences | TR (ms) | TE (ms) | imaging matrix (mm ×mm) | slice thickness (mm) |
| --- | --- | --- | --- | --- | --- |
| **Siemens SKYRA** | T1WI | 2457 | 13 | 240×240 | 5 |
|  | T2 FLAIR | 9553 | 165 | 240×240 | 5 |
| **Siemens VERIO** | T1WI | 1900 | 2.52 | 250×250 | 1 |
|  | T2 FLAIR | 8000 | 110 | 256×256 | 6 |
| **GE Signa** | T1WI | 2600 | 13.2 | 240×240 | 5 |
|  | T2 FLAIR | 8500 | 81 | 240×240 | 5 |
| **Siemens Avanto** | T1WI | 2000 | 9 | 240×240 | 5 |
|  | T2 FLAIR | 9000 | 119 | 250×250 | 4 |

**Supplementary table1** The comparison of feature selection methods
